# Evaluation of Dispersion Methods and Mechanical Behaviour of Glass Fibre Composites with Embedded Self-Healing Systems

**DOI:** 10.3390/polym13101642

**Published:** 2021-05-18

**Authors:** Ionut Sebastian Vintila, Sorin Draghici, Horia Alexandru Petrescu, Alexandru Paraschiv, Mihaela Raluca Condruz, Lucia Raluca Maier, Adela Bara, Madalina Necolau

**Affiliations:** 1 National Research and Development Institute for Gas Turbines COMOTI, 061126 Bucharest, Romania; alexandru.paraschiv@comoti.ro (A.P.); raluca.condruz@comoti.ro (M.R.C.); raluca.maier@comoti.ro (L.R.M.); 2 Advanced Polymer Materials Group, University Politehnica of Bucharest, 011061 Bucharest, Romania; madalinanecolau@gmail.com; 3 Laboratory for Advanced Testing and Mechanical Characterization of Materials, Department of Strength of Materials, Faculty of Industrial Engineering and Robotics, University Politehnica of Bucharest, 060042 Bucharest, Romania; sorin.draghici@upb.ro (S.D.); horia.petrescu@upb.ro (H.A.P.); 4 National Institute for Research and Development in Electrical Engineering ICPE-CA Bucharest, 030138 Bucharest, Romania

**Keywords:** polymer composites, self-healing, thermal stability, FEM analysis, dicyclopentadiene, 5-ethylidene-2-norbornene

## Abstract

The present paper is focused on evaluating the most suitable dispersion method in the epoxy matrix of two self-healing systems containing dicyclopentadiene (DCPD) and 5-ethylidene-2-norbornene (ENB) monomers encapsulated in a urea-formaldehyde (UF) shell, prior to integration, fabrication and impact testing of specimens. Both microstructural analysis and three-point bending tests were performed to evaluate and assess the optimum dispersion method. It was found that ultrasonication damages the microcapsules of both healing systems, thus magnetic stirring was used for the dispersion of both healing systems in the epoxy matrix. Using magnetic dispersion, 5%, 7%, 10%, 12% and 15% volumes of microcapsules were embedded in glass fibre composites. Some of the samples were subjected to thermal cycling between −20 °C and +100 °C for 8 h, to evaluate the behaviour of both healing systems after temperature variation. Impact test results showed that the mechanical behaviour decreases with increasing microcapsule volume, while for specimens subjected to thermal cycling, the impact strength increases with microcapsule volume up to 10%, after which a severe drop in impact strength follows. Retesting after 48 h shows a major drop in mechanical properties in specimens containing 15% MUF-ENB microcapsules, up to total penetration of the specimen.

## 1. Introduction

Thermoset polymers are widely used as matrices in most fibre-reinforced polymers (FRP) composites used in structural applications due to their ease of processing, low cost and good wettability. They also offer good mechanical properties, as well as good corrosion resistance compared to metals and other engineering materials. They are subjected to different types of stresses during service life, and due to their uniqueness, they have different failure mechanisms compared to metals. Crack detection and repair in these types of materials are difficult and, therefore, self-healing techniques have been explored in a variety of ways to overcome this limitation. Another important issue is concerned with the durability of these materials as they are exposed to different service loading due to their viscoelastic properties [1,2,3].

Epoxy resins are widely used in industrial applications due to their excellent chemical, physical and mechanical properties. Epoxy could be one of the more effective materials as a healing agent over other available healing compounds due to its cost and healing efficiency for self-healing composites. The incorporation of the capsules into the epoxy matrices is mainly achieved through dispersion processes where the capsule shell shall be strong enough to sustain the stirring forces. Shukla et al. [4] used ultrasonication as a method to disperse microencapsulated DCPD healing systems into the epoxy matrix, using different concentrations of microcapsules (0.5, 1.0 and 1.5 wt.%). The corresponding amount of microcapsules were firstly mixed with acetone and sonicated for one hour followed by the addition of epoxy resin and sonicated again for one hour. It was found that the fracture toughness of composites increased with the increase in wt.% of microcapsules, namely, an improvement of 78% in virgin fracture toughness was observed for 1.5 wt.% microcapsules with an increase of 84%, 75% and 68% at 0.5, 1 and 1.5 wt.% of microcapsules, respectively, in comparison to that of neat epoxy after healing. In another study, Blaiszik et al. [5] dispersed microcapsules using ultrasonication and high-speed stirring in an epoxy matrix of EPON 828 (DGEBA) resin cured with Ancamine (DETA). A slight decrease in the elastic modulus along with a more significant decrease in ultimate tensile strength was measured for composites with included capsules with a mean diameter of 1.5 µm. The decrease in tensile strength was accompanied by a significant increase in fracture toughness. Epoxy with dispersed sonicated capsules showed a nearly 59% increase in fracture toughness for a capsule volume fraction of 0.015.

Because of their heterogeneous nature and construction, composite materials have improved mechanical properties [6,7], but the propagation of a crack is more complex than that in homogeneous materials. The fracturing of a composite involves the breaking of the load-bearing fibres and the weak matrix, and a complex mixture of crack deviances along these weak interfaces [8,9]. The choice of resins and fibres, the manner of combination in the composite, and the quality of the industrially made composite should all be judiciously organised if ideal toughness is to be achieved. Low-velocity impact damage is one of the significant concerns for composite materials. Composite materials show good resistance if the applied load is in the fibre direction, but lower resistance in the transverse direction. Matrix-fibre debonding, delamination, matrix micro-cracking and fibre pull out are the common failure modes in composites under impact loading. Motuku et al. [10] studied the effect of low-velocity impact on self-healing materials. Their experiments showed that the insertion of glass capillary pipettes as storage tubes did not alter the impact response of the composites, and the healing agents could be released in the case of cracking. Bleay et al. [11] investigated the compression performance of glass fabric self-healing composites after low-velocity impact. When the impact-treated specimens were exposed to a combination of high temperature and vacuum, an improvement of almost 10% in compression strength was observed. Yin [12] measured compression after impact CAI of woven fibreglass-reinforced epoxy composites impacted up to 3.5 J, showing improved healing performance at lower impact energies and through the application of lateral pressure.

Patel et al. [13] studied the low-velocity impact on the glass fibre-reinforced composites with dicyclopentadiene (DCPD)-loaded microcapsules and paraffin wax microspheres containing 10 wt.% Grubbs’ catalysts. About 51% of crack length was reduced due to the healing efficiency after low-velocity impact. Williams et al. [14] investigated the healing efficiency of a carbon fibre-reinforced composite (CFRP) with hollow glass fibres embedded as healing agent containers. The healing efficiency was assessed based on compression testing after low-velocity impact and they reported a 90% recovery for this system.

Zainuddin et al. [15] studied the effect of the healing recovery for glass fibre-reinforced composite. They used a self-healing agent embedded in hollow glass tubes aiming for the improvement of peak load in low-velocity impact (LVI) testing. The LVI experiment was performed at 56 J for all specimens and they registered a 53.6% improvement after the second impact for the self-healing composite in comparison to reference specimens. Hosur et al. [1] studied the extrinsic self-healing concept using urea-formaldehyde microcapsules containing room temperature curing epoxy resin system as the healing agent prepared by in situ polymerization. The healing performance was measured in terms of a low-velocity impact test with 30 J and 45 J impact loads. Results analysis confirmed the delamination and micro crack in composite materials and subsequent healing recovery observed in terms of damaged area reduction and restoration of mechanical properties.

During high-velocity impacts, characterised in the range of 300–2500 ms^−1^ [16,17], the event is so short that the structure has no time to respond in global flexural or shear modes and so damage tends to be localised, taking the form of target penetration. During low-velocity impacts (generally regarded to be at velocities less than 10 ms^−1^), the impactor contact time is long enough for the entire FRP structure to respond to the impact [18]. The out of plane displacement generates flexural and shear stresses that, in the main, lead to cracking of the matrix material, although fracture of fibres can also occur.

Trask et al. [19] characterised damage formation and self-healing efficiency within vascularised CFRP laminates over a range of low-velocity impact energies and studied the ability to deliver a healing agent to regions of impact-induced damage via an embedded vasculature thus restoring a proportion of the undamaged material properties. After impact, non-destructive evaluation (NDE) analysis highlighted that vascularised laminates follow the same damage size to residual compression strength relationship as found for conventional laminates. A damage size greater than 14.9 mm was also found to cause a shift in failure mode from end brooming to central buckling and collapse, with an accompanying significant decrease in residual compression strength. This data is crucial for the design of self-healing laminates/components as it provides a quantifiable metric for vascular spacing.

The impact of the microcapsule estimate on the mechanical properties is a blend of the closeness of the microcapsule itself, which expands the mechanical quality with expanding size, and also the self-mending impact [20,21]. The wide assortment of studied shell structures for similar properties, notwithstanding, implies that a portion of these outcomes must be taken with care [22].

The objective of this paper is to give an overview of the differences between the two most used dispersion methods, namely dispersion by magnetic stirring and dispersion by sonication, and their effect on the self-healing microcapsules when embedded in the host epoxy matrix. Their dispersion effect was investigated by both microstructural analyses and by three-point bending tests. Consequently, glass fibre impact specimens were fabricated with varying microcapsule volume and tested to evaluate their impact strength with respect to microcapsule addition.

## 2. Materials and Methods

The materials used for the synthesis of poly-urea-formaldehyde (PUF) microcapsules containing dicyclopentadiene (DCPD) as a healing agent are presented in Table 1 while Table 2 presents the materials used for the synthesis of melamine-urea-formaldehyde (MUF) microcapsules containing 5-ethylidene-2-norbornene (ENB) as a healing agent. Materials presented in Table 1 and Table 2 were purchased from Sigma Aldrich (Sigma-Aldrich Chemie Gmbh, Munich, Germany).

Prior to the integration of self-healing systems in the glass fibre-reinforced epoxy matrix and evaluation of their impact mechanical properties, an investigation was conducted to find the optimal dispersion method. E-glass fibre weave (purchased from Raymond group, Vsetín, Czech Republic) with a specific weight of 280 g/m^2^ was used as a reinforcement for the epoxy matrix. Resoltech 1050/1055S (Resoltech SAS, Rousset, France) epoxy system was used to embed the self-healing agents and the behaviour of the two different dispersion methods was observed.

### 2.1. Dispersion by Sonication

Before the integration of the self-healing systems in the epoxy matrix, sonication parameters were established, varying the amplitude, pulsation time, off time and total sonication time, using a VIBRA CELL VCX 130 (Sonics & Materials Inc., Newtown, CT, USA) with a CV18 probe. Samples were cured at 80 °C for 120 min and microstructural analysis was conducted to observe the best sonication parameters (Table 3).

It was concluded that the best sonication parameters for the dispersion of self-healing microcapsules in the epoxy matrix were at an amplitude of 40%, 10 s pulsation with 2 s off time and a total sonication time of 10 min.

### 2.2. Dispersion by Magnetic Stirring

As concluded in [23], a homogenous dispersion by magnetic stirring was found at a speed of 100 rpm and a temperature of 60 °C using an IKA C-MAG MS 10 (Sigma-Aldrich Chemie GmbH, Taufkirchen, Germany) magnetic stirrer with ETS-D6 sensor.

### 2.3. Fourier Transform Infrared Spectroscopy Analysis

As the PUF-DCPD and MUF-ENB microcapsules batch were the same as in [23] and kept in sealed flasks for several months, a Fourier transform infrared (FT-IR) analysis was required to validate the presence of the core material. The analysis was performed on a Bruker VERTEX 70 (Bruker Optik GmbH, Leipzig, Germany) instrument employing 32 scans in the range of 400–4000 cm^−1^ and equipped with attenuated total reflection (ATR) using a Ge crystal.

### 2.4. Thermogravimetric Analysis

Thermal analysis was also performed on the two self-healing systems, consisting of thermogravimetric analysis (TGA) and derivative thermogravimetry (DTG), to observe if their physical properties were altered. TGA analysis was completed on a TG 209 F1 Libra instrument (NETZSCH-Gerätebau GmbH, Selb, Germany) using a sample of about 5 mg. The sample was heated in the temperature range 20–700 °C with a heating rate of 10 ± 0.2 °C/min in N_2_ atmosphere using a Platinum/Rhodium crucible.

### 2.5. Dynamic Mechanical Analysis

Dynamic mechanical analysis (DMA) analysis was recorded on a TRITEC 2000 B instrument (Triton Technology Ltd., Nottinghamshire, UK). The samples were analysed in the single cantilever bending mode, subjected to 1 Hz force and heated in a temperature range of 25–180 °C, with a heating rate of 5 ± 0.2 °C/min.

### 2.6. Fabrication of Testing Specimens

#### 2.6.1. Three-Point Bending Specimens

Neat epoxy reference specimens were fabricated according to SR EN ISO 14125:2003 (Class IV) and were compared with specimens containing PUF-DPCD and MUF-ENB self-healing systems, integrating into the epoxy matrix by means of both dispersion methods. This test was conducted to observe the mechanical behaviour of the two dispersion methods. An amount of 5% microcapsules were embedded in the epoxy matrix and cured at 80 °C for 120 min.

#### 2.6.2. Impact Specimens

Glass fibre-reinforced polymer (GFRP) specimens were fabricated according to ISO D7136/D7136M-07 standard, by means of the magnetic stirring dispersion method. Specimens were cured at 80 °C for 120 min. A total of 25 impact test specimen were fabricated (Table 4), among which, 5 specimens were subjected to thermal cycling, from −20 °C to +100 °C for a period of 8 h, to evaluate the behaviour of the self-healing system after thermal exposure. Because the impact test was performed at a relatively low force (50 J), only to observe the repair mechanism not to determine the mechanical properties of the composite material, the resin-microcapsule-catalyst mixture will be applied only between the first three layers. All specimens were cut with a water jet to final dimensions.

### 2.7. Specimen Testing

Three-point bending tests were performed with the Instron 3360 Series Universal Testing instrument (Instron, MA, USA), with a force cell of 50 kN.

In order to perform the impact tests, a series of preliminary tests were conducted to identify the impact energy, energy that must not cause the penetration of the specimen or leave a very strong impact mark. Thus, the optimal impact energy of 50 J was identified using Equation (1). Drop height and impact velocity were calculated using Equations (2) and (3). Dispersion by sonication was not considered during this test, as the method does not provide sufficient homogenization of the two self-healing systems in order to investigate the impact behaviour and healing process evaluation. The impact mechanical tests were performed on an Instron 9340 Series Universal Testing instrument (Instron, Norwood, MA, USA), with a force cell of 20 kN.

(1) $$E=C_{\varepsilon }\times \;h$$

where E = potential energy of impactor prior to drop [J], C_ε_ = specific ratio of the impact energy to specimen thickness [6.7 J/mm], h = nominal thickness of the specimen [mm].

(2) $$H=\frac{E}{m_{d}\times \;g}$$

where H = drop-height of impactor [m], m_d_ = mass of the impactor for drop height calculation [kg], g = acceleration due to gravity [9.81 m/s^2^].

(3) $$\nu_{i}=\frac{W_{12}}{t_{2}-t_{1}}+g\left(t_{i}-\frac{t_{1}+t_{2}}{2}\right)$$

where ν_i_ = imact velocity [m/s], W_12_ = distance between leading edges of first (lower) and second (upper) flag prongs [m], t_1_ = time first (lower) flag prong passes detector [s], t_2_ = time second (upper) flag prong passes detector [s], t_i_ = time of initial contact obtained from force vs. time curve [s].

### 2.8. Tridimensional Analysis

Following the impact tests on the specimens containing self-repair systems, three-dimensional analyses were performed on an Altera 10.10.08 measuring equipment (NIKON, Tokyo, Japan) with PH10M PLUS probe indexing head in order to observe in detail the impact area and its depth.

## 3. Results

### 3.1. Thermal Mechanical Behaviour

**Figure 1:**
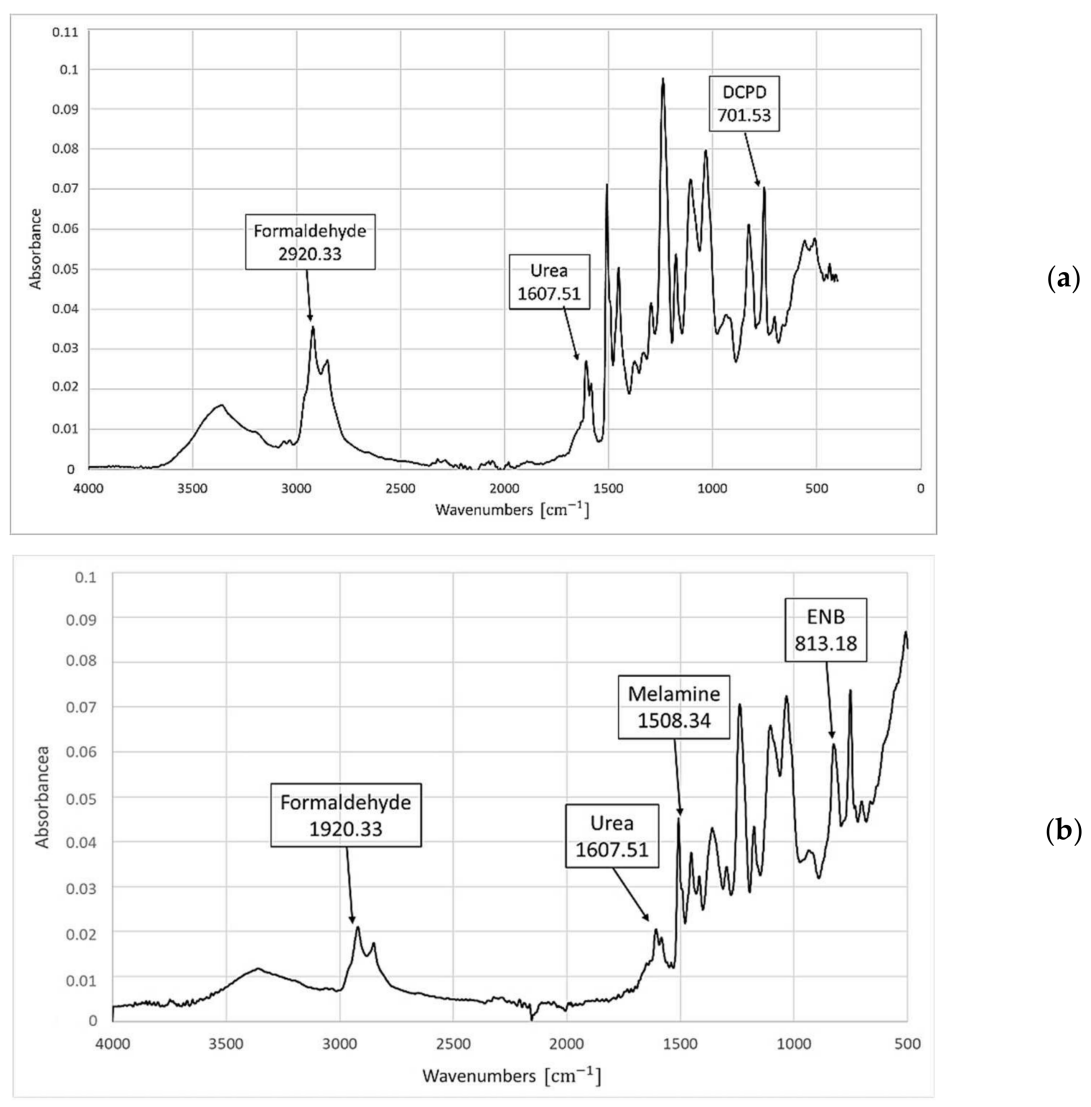
FT-IR analysis of (**a**) PUF-DCPD and (**b**) MUF-ENB self-healing systems.

The FT-IR analysis is presented in Figure 1 and confirms the presence of the core materials. The spectra confirm that the shell material of the capsules containing poly-urea-formaldehyde polymer and melamine-urea-formaldehyde polymer is present. Furthermore, the FT-IR spectra of DCPD and ENB have been identified proving the preservation of both DCPD and ENB healing systems in the UF/MUF shell. It was, therefore, concluded that both systems contain the core and shell corresponding peaks.

**Figure 2:**
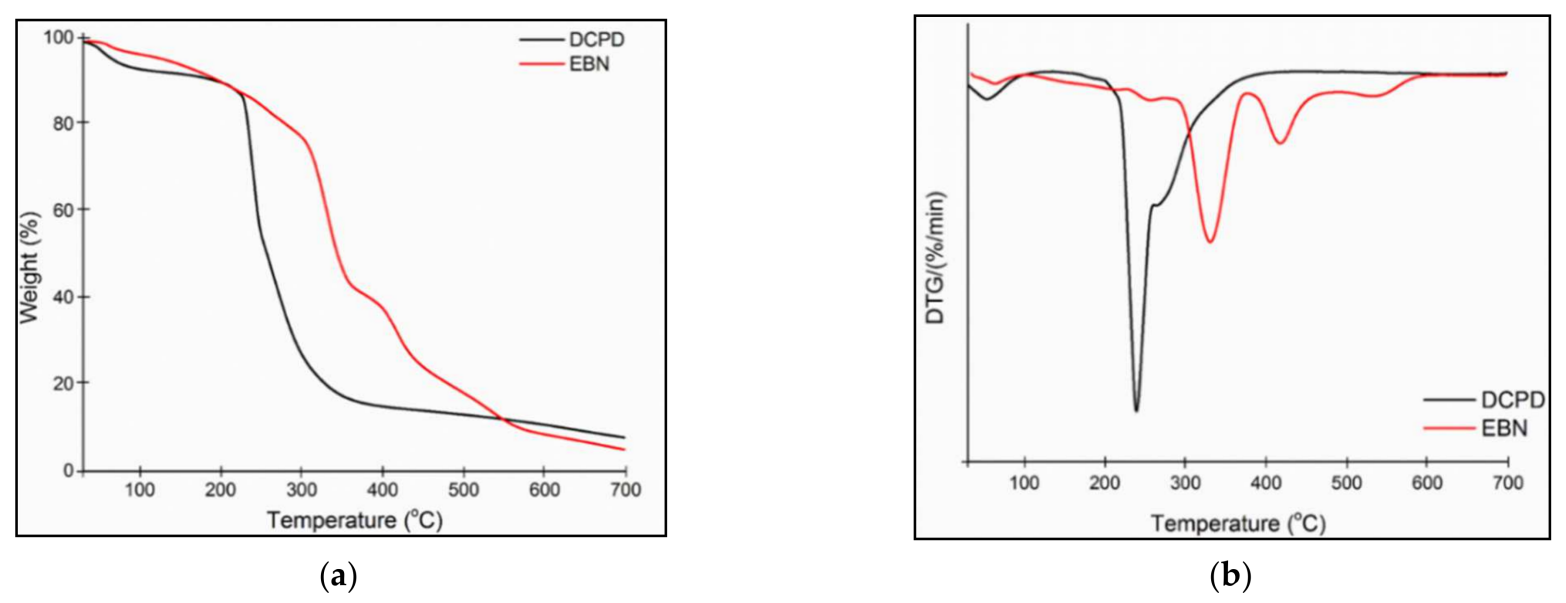
(**a**) TGA and (**b**) DTG analysis of PUF-DCPD and MUF-ENB systems.

From the TGA analysis presented in Figure 2, it was also concluded that the PUF-DCPD system is thermally stable up to 200 °C after which thermal degradation can be observed up to 450 °C. As for the MUF-ENB system, thermal stability was observed until 250 °C after which a continuous thermal degradation was recorded. A total mass loss of approximately 80% was found for the PUF-DCPD system and approximately 82% for the MUF-ENB system.

**Figure 3:**
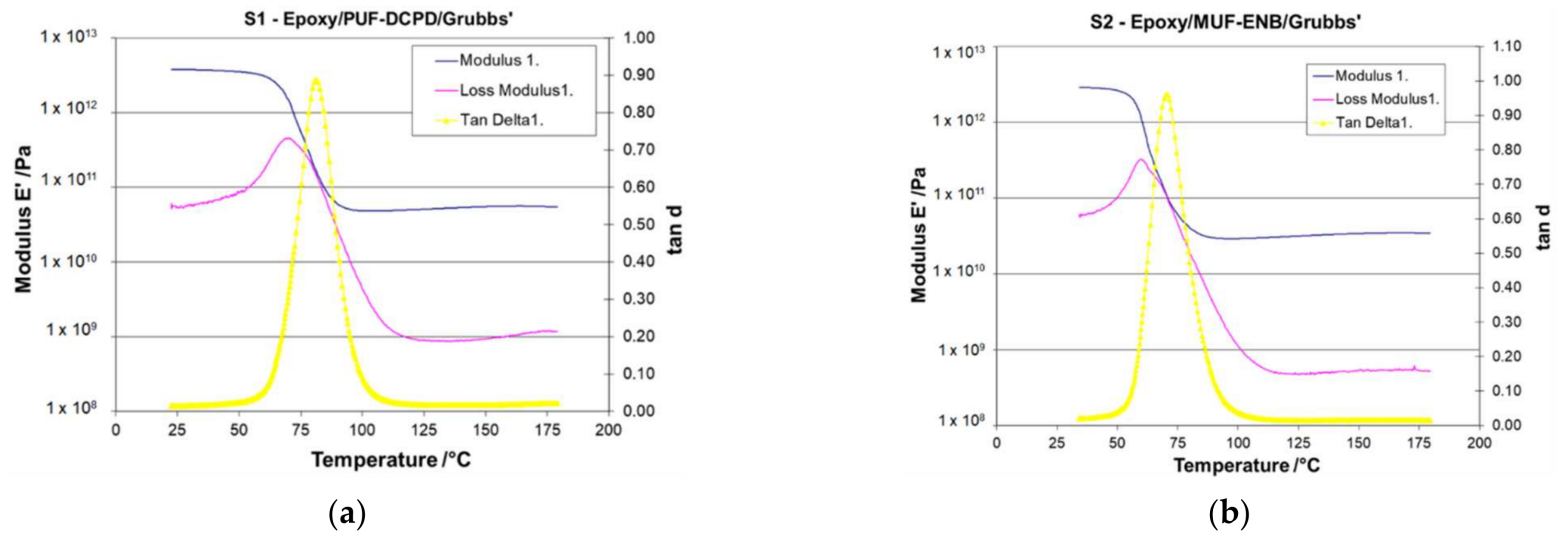

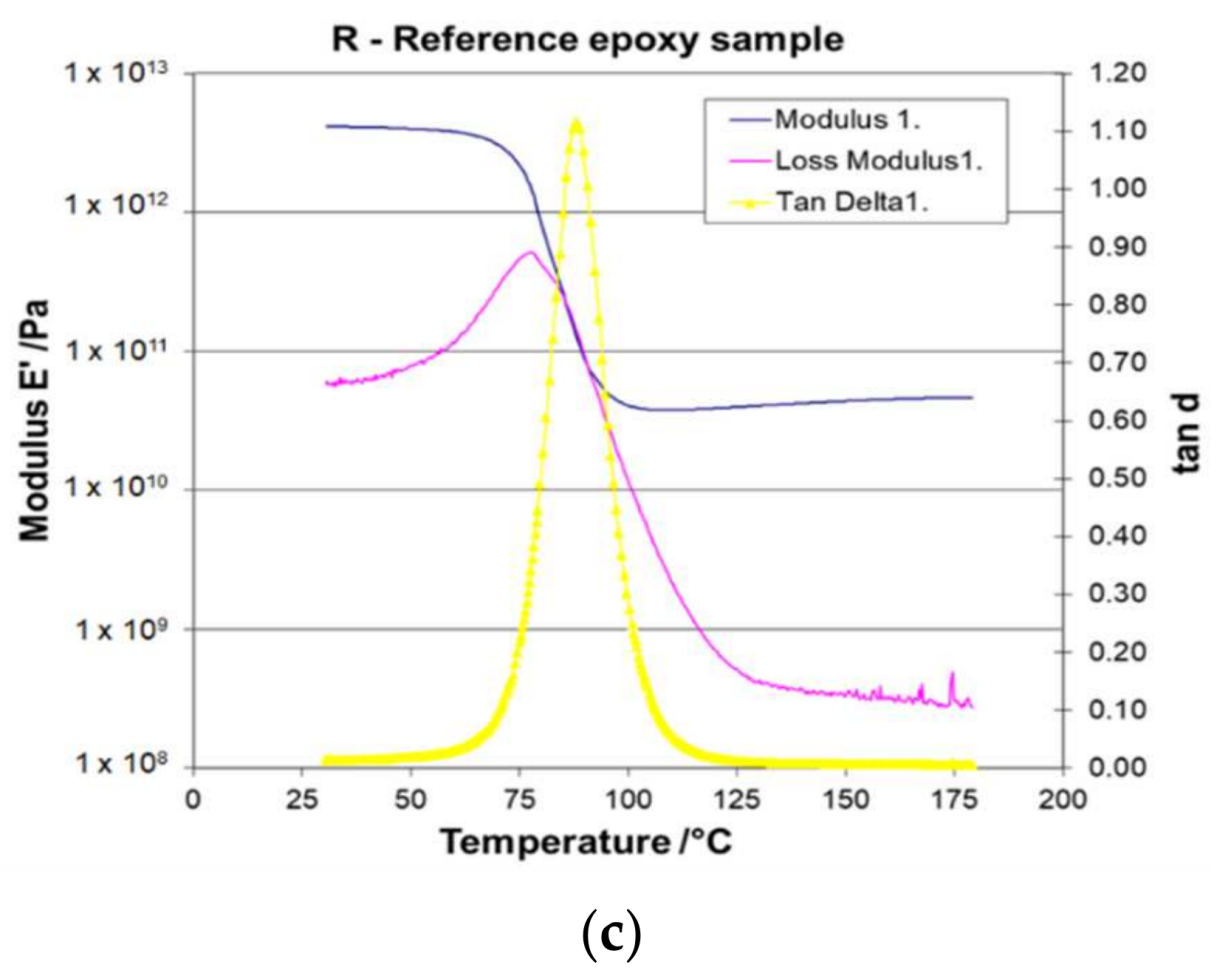
DMA analysis of (**a**) PUF-DCPD, (**b**) MUF-ENB self-healing systems and (**c**) neat epoxy matrix.

Along with the incorporation of the microcapsules into the polymeric matrix, a decrease in the mechanical properties occurs as described by Figure 3. The value for the crosslinking density of the S1 sample (Table 5) suggests that there are some interactions between the DCPD and epoxy that participate in the network formation. The microcapsules interfere in the microstructure of the polymeric material by hindering the reactants or by generating agglomerates. The crosslinking density was calculated according to the method proposed by Hill [24]. Figure 3 shows the DMA curves (Storage modulus, loss modulus and tan δ) for the pure epoxy sample along with the two self-healing systems containing DCPD and ENB. The first area of the DMA curves appears at lower temperatures and relates to the glassy region, where the material presents as a rigid solid. The second region exhibits a severe decrease of the storage modulus associated with the glass transition region. The last region consists of the rubbery plateau where the storage modulus accedes in a steady-state condition due to the fact that the material enters into a soft rubber state. The curves for the storage modulus (E′) are related to the elastic modulus associated with the response of the materials to a viscoelastic deformation and is often associated with the stiffness of the material.

**Scheme 1:**
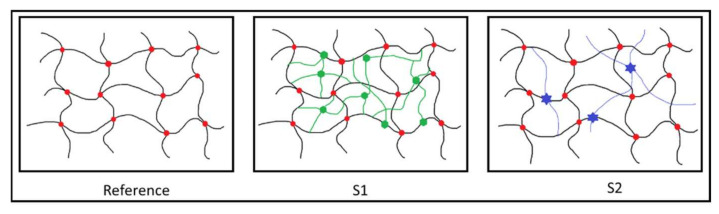
Schematic representation of the crosslinking density related to the self-healing microcapsules.

The epoxy reference sample displays an intermediate value of E′ in comparison with those containing the self-healing systems. A reduced value of the storage modulus for S1 suggests a stiffer material, as sustained also by the increased value of the crosslinking density. This may be a consequence of the urea microcapsules breaking and releasing the self-healing monomers. The S2 sample has a more elastic behaviour as suggested by the increased value of the storage modulus. However, although there is a small difference between the crosslinking density values compared to the reference sample, the variation of the Tg values is significant. In comparison with the neat epoxy sample, the crosslinking density of the two self-healing systems increased as a consequence of the participation of the self-healing monomers within the urea microcapsules in the network formation (as seen in Scheme 1). Usually, a high value for the Tg is usually accompanied by an increased value of the crosslinking density and an increase in the crosslinking density suggests a reduction of the free volume of the macromolecules. The different behaviour of the self-healing systems may be a consequence of the structural differences between the monomers used.

### 3.2. Microstructural Analysis

**Figure 4:**
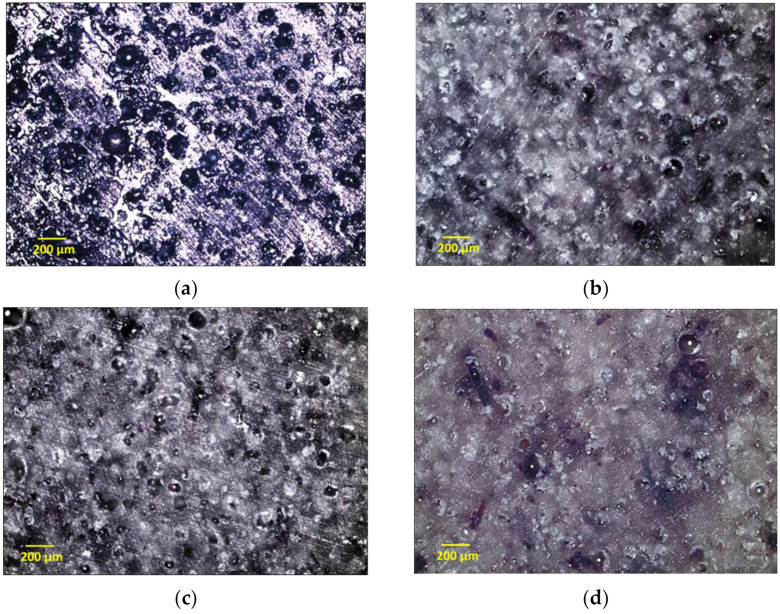
Optical images illustrating the dispersion by (**a**) magnetic stirring and (**b**) sonication of PUF-DCPD system and dispersion by (**c**) magnetic stirring and (**d**) sonication of MUF-ENB system.

**Figure 5:**
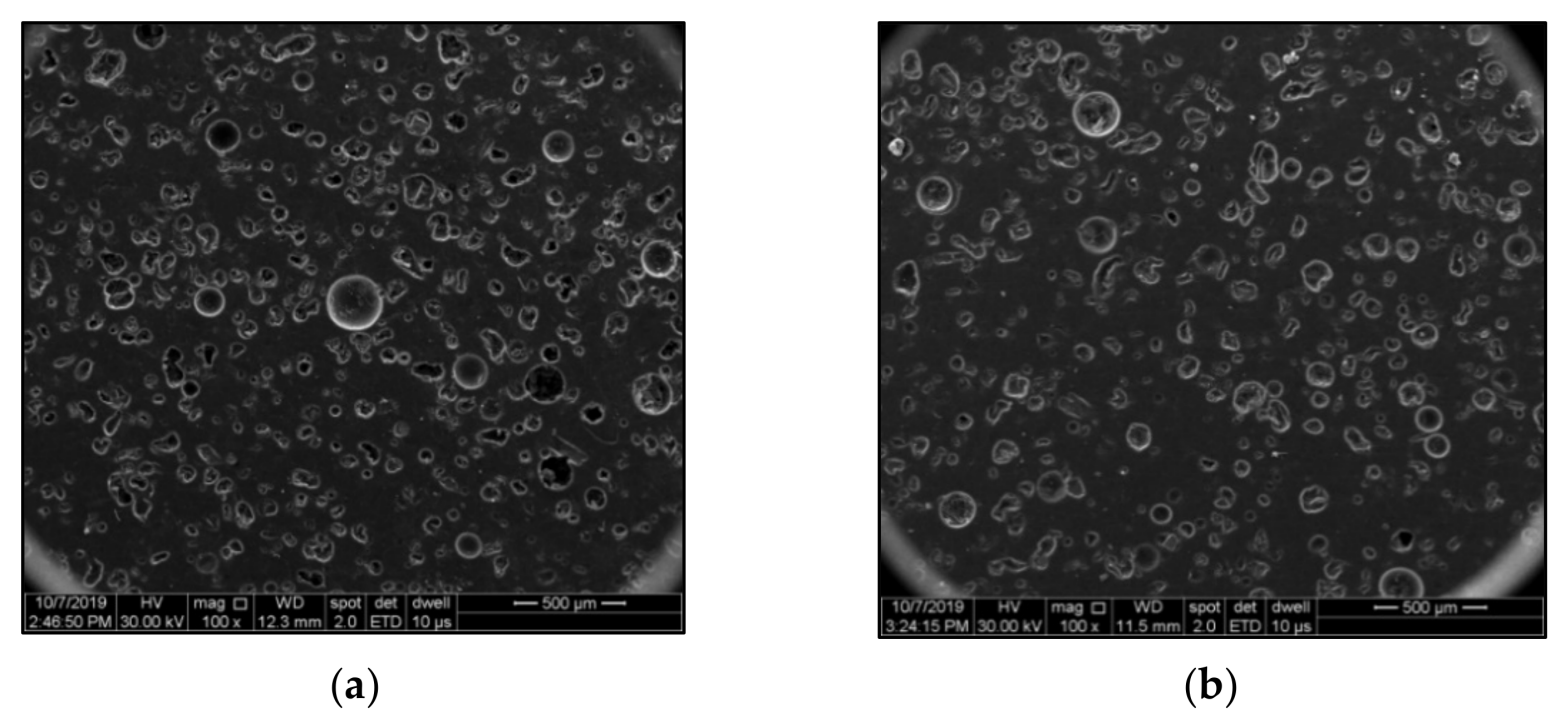

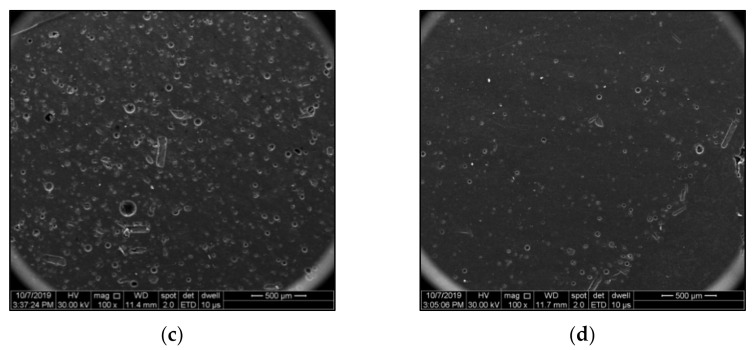
SEM images illustrating the dispersion by (**a**) magnetic stirring and (**b**) sonication for the PUF-DCPD system and dispersion by (**c**) magnetic stirring and (**d**) sonication for the MUF-ENB system.

**Figure 6:**
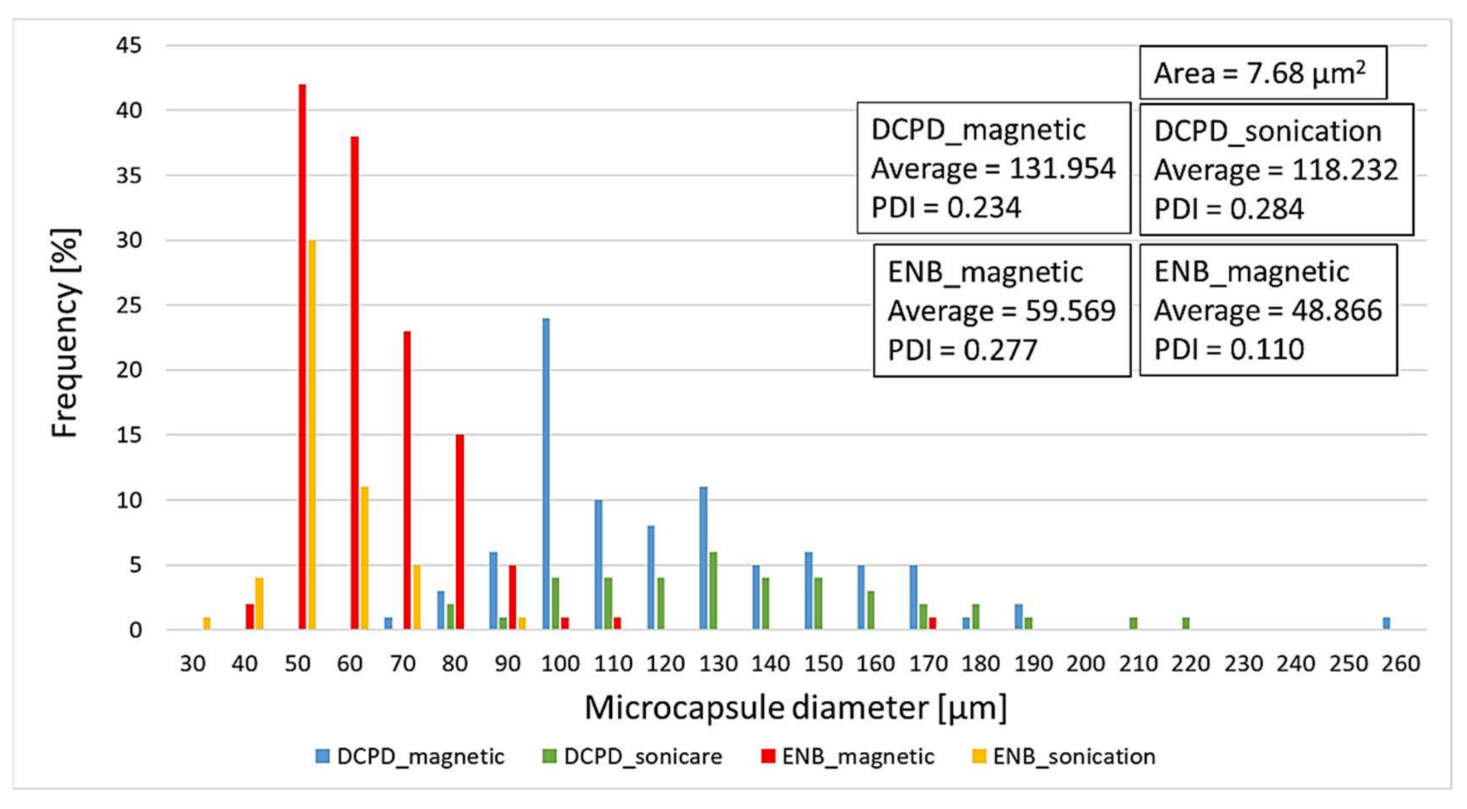
Statistical calculation of microcapsule size.

Both light optical and scanning electron microscopy (SEM) analysis was conducted to observe and compare the two dispersion methods using Inspect F50 equipment (FEI, Hillsboro, OR, USA). The images taken from the optical microscope (Figure 4a–d) showed a better homogenisation process for the magnetic stirring dispersion method. The SEM analysis also confirms the better homogenisation of the two self-healing systems by magnetic stirring compared to sonication (Figure 5a–d). Due to the small dimensions of the MUF-ENB microcapsules, they tend to break when subjected to sonication. Particle size distributions were measured using Scandium software, version 5.2 (Olympus Soft Imaging Solutions GmbH, Münster, Germ), on 4 different SEM images and are presented in Figure 6. Mean diameters of 131.95 µm and 118.23 µm were found for DCPD microcapsule dispersed by magnetic stirring and sonication, respectively, while for ENB microcapsules, a mean diameter of 59.57 µm was found for magnetic dispersion and 48.86 µm for sonication.

It is known that DCPD microcapsules have a larger diameter (150–250 µm) compared to ENB microcapsules (30–100 µm). During sonication, despite the variation of amplitude and pulsation, both microcapsule systems were damaged. Microcapsules with smaller diameters are most prone to damage for both systems despite the variation of amplitude and pulsation while larger microcapsules are less prone to damage, as the larger microcapsules can sustain larger deformations with less strain.

### 3.3. Specimen Testing

#### 3.3.1. Three-Point Bending Specimens

**Figure 7:**
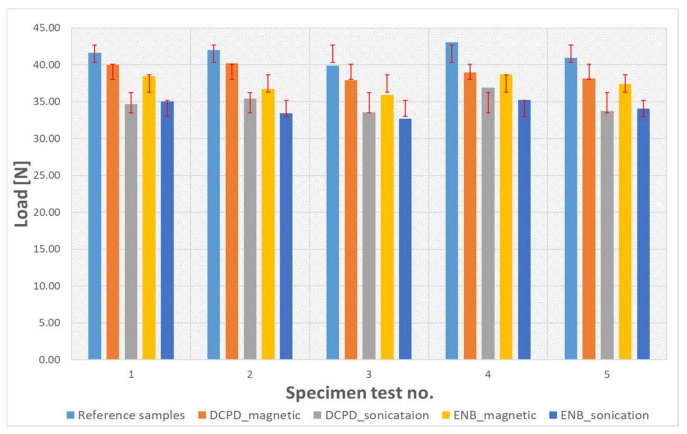
Load values for the three-point bending tested specimens.

**Figure 8:**
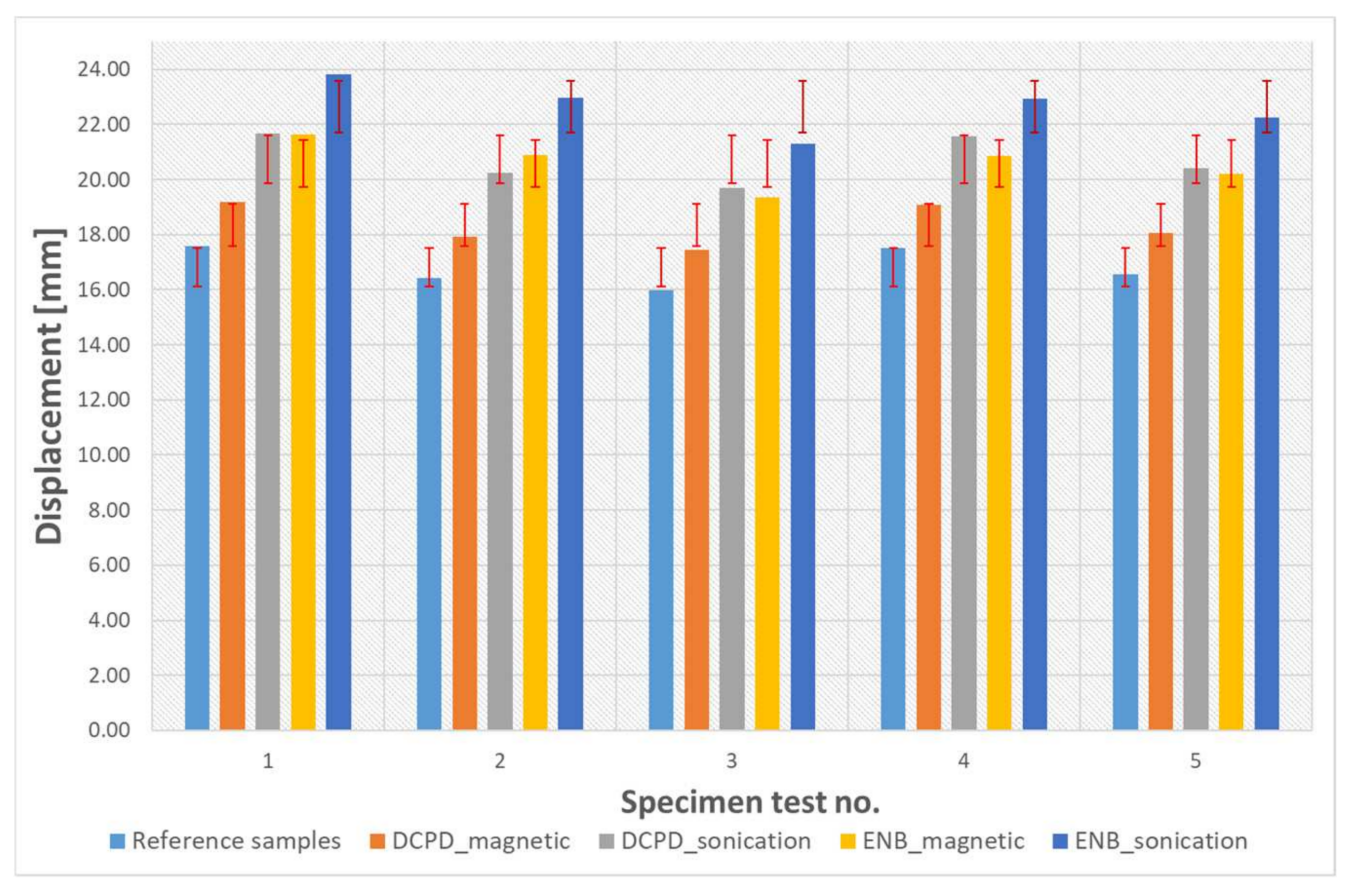
Displacement values for the three-point bending tested specimens.

The load was applied to the specimens at mid-span until rupture. Table 6 presents the flexural stress at break values for the reference specimens and specimens with embedded microcapsules following the two dispersion methods, and Table 7 presents the three-point bending flexural modulus and calculated absorbed energy. Due to the large number of tested specimens and to obtain a better understanding of the results, the load-displacement curves are presented as two separate charts, as seen in Figure 7 and Figure 8. With respect to the values in Table 7, it is known that adding any rigid or elastic particles to polymers tends to decrease the mechanical properties of such polymers [25,26]. It can be also explained that both systems shell materials (urea-formaldehyde and melamine-urea-formaldehyde) are compatible with epoxy, creating a solid interfacial interaction during curing. However, as the dispersion method by magnetic stirring was found here to be the optimal solution by maintaining a considerable volume of microcapsules compared to the sonication method, as seen from Figure 5, and due to the elastic behaviour of the microcapsules, the specimens containing microcapsules tend to have a more elastic behaviour than reference specimens, and translates into a decrease of flexural modulus. Reference epoxy specimens absorb more energy during load as compared to DCPD and ENB specimens due to their microcapsules elastic behaviour. As the microcapsule volume fraction is higher for the magnetic dispersion, its absorbing energy is higher compared to sonication dispersions.

Similar to the microstructural analysis of the two dispersion methods, it was also observed from the three-point bending tests that dispersion by sonication reduces the flexural strength of the DCPD specimens by 17% and by 20% for the ENB specimens, compared to magnetic stirring, which has a drop in flexural strength of 9% for DCPD specimens and of 11% for the ENB specimens. As also seen in Table 3 and Figure 5, using the corresponding sonication parameters tends to destroy most of the microcapsules during homogenization, releasing the monomer that, without the presence of a catalyst, does not take part in the curing process and thus reduces the mechanical properties of the specimens. Considering the magnetic dispersion of both systems, the values can be considered acceptable, as these microcapsules are considered an induced defect.

Moreover, specimens containing the PUF-DCPD system showed an average displacement of 9% for magnetic stirring dispersion and 15% for sonication, whilst for MUF-ENB specimens, magnetic stirring dispersion showed a displacement of 22% and 35% for sonication, when compared to neat epoxy specimens. This may be due to the considerably smaller dimensions of MUF-ENB microcapsules (<100 µm) when compared to PUF-DCPD microcapsules (approx. 250 µm). Specimens were tested until the load was constant, which is the starting point of material degradation.

#### 3.3.2. Impact Specimens

**Figure 9:**
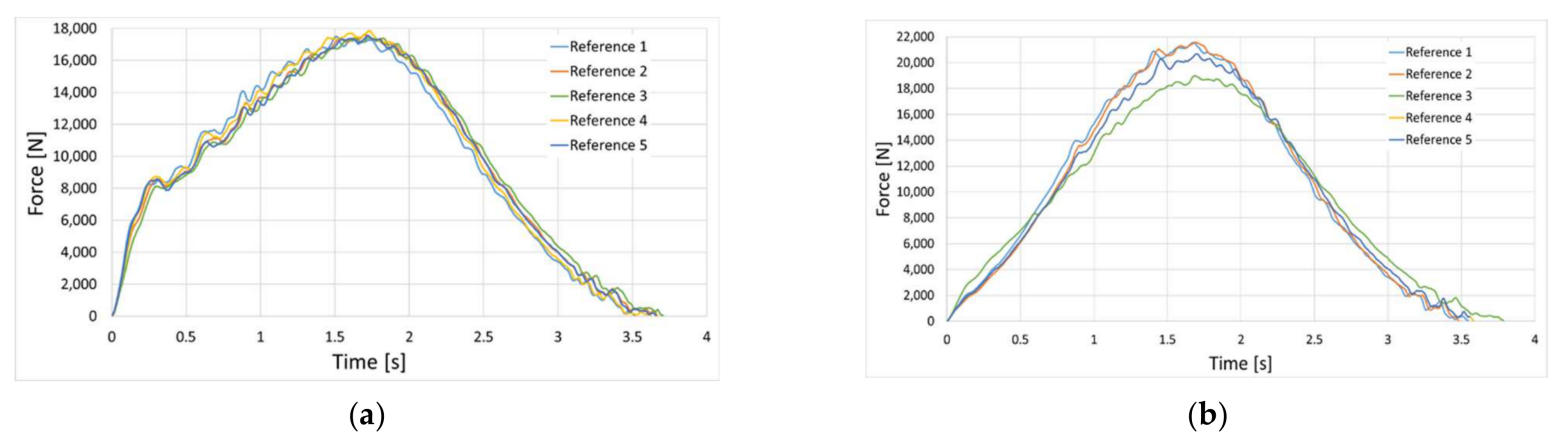

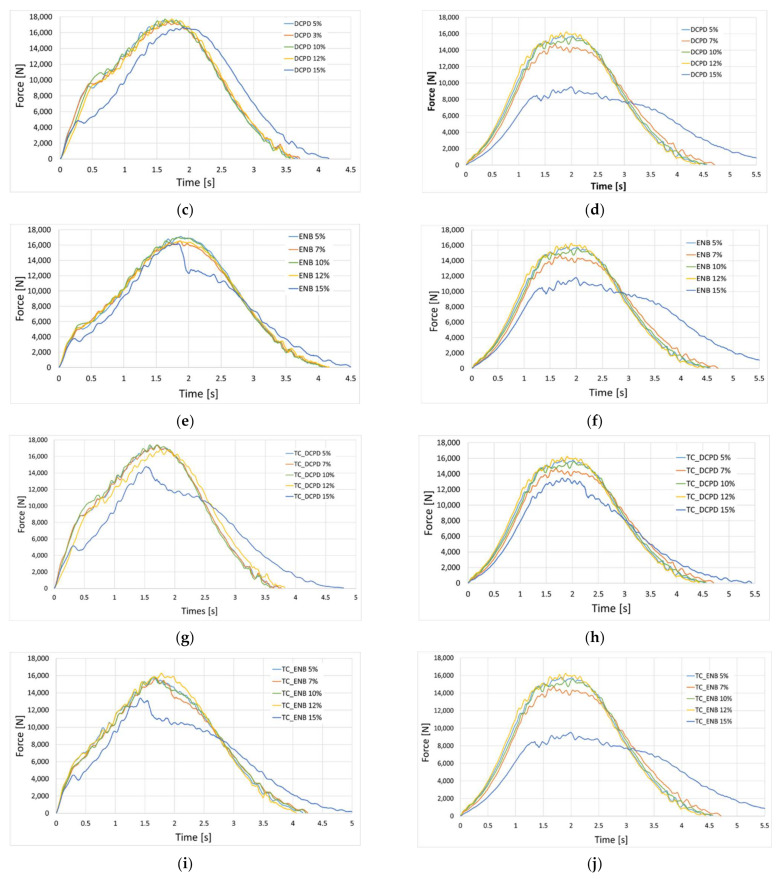
Impact test results after first and second test for (**a**,**b**) reference, (**c**,**d**) PUF-DCPD, (**e**,**f**) MUF-ENB, (**g**,**h**) PUF-DCPD after thermal cycling and (**i**,**j**) MUF-ENB after thermal cycling specimens.

All impact specimens were subjected to 50 J impact energy. After the first impact, all specimens were introduced in an oven at 40 °C in ambient atmosphere, left for 48 h, and retested in the same conditions. The impact strength with respect to microcapsule addition is presented below, in Figure 9.

As can be seen from the test results in Figure 9, increasing the microcapsule volume has little to no effect on the impact force; however, subjecting the specimens to temperature variation from −20 °C to +100 °C indicated a decrease in impact force, especially for the 15% microcapsule addition, for both healing systems. After retesting, specimens containing the PUF-DCPD healing system present a smaller decrease of impact force as well as a difference in the impact behaviour for the specimen containing 15% microcapsule volume. This may indicate that a high volume of microcapsules (on a small area) cannot lead to the repair of the composite material, which acts as a defect and causes significant reductions in the mechanical properties of the composite material. Retesting of MUF-ENB specimens shown a similar impact force as PUF-DCPD specimens, but a smaller decrease for specimen with 15% microcapsules. This may indicate that the MUF-ENB repair system performs better when using a high volume of microcapsules on a small surface. For the specimens subjected to thermal cycles and retested, the major difference was observed in the case of the specimens with the largest volume of microcapsules. However, this time the specimen containing 15% PUF-DCPD microcapsules responded better to the impact loads compared to the specimens containing 15% MUF-ENB microcapsules.

**Figure 10:**
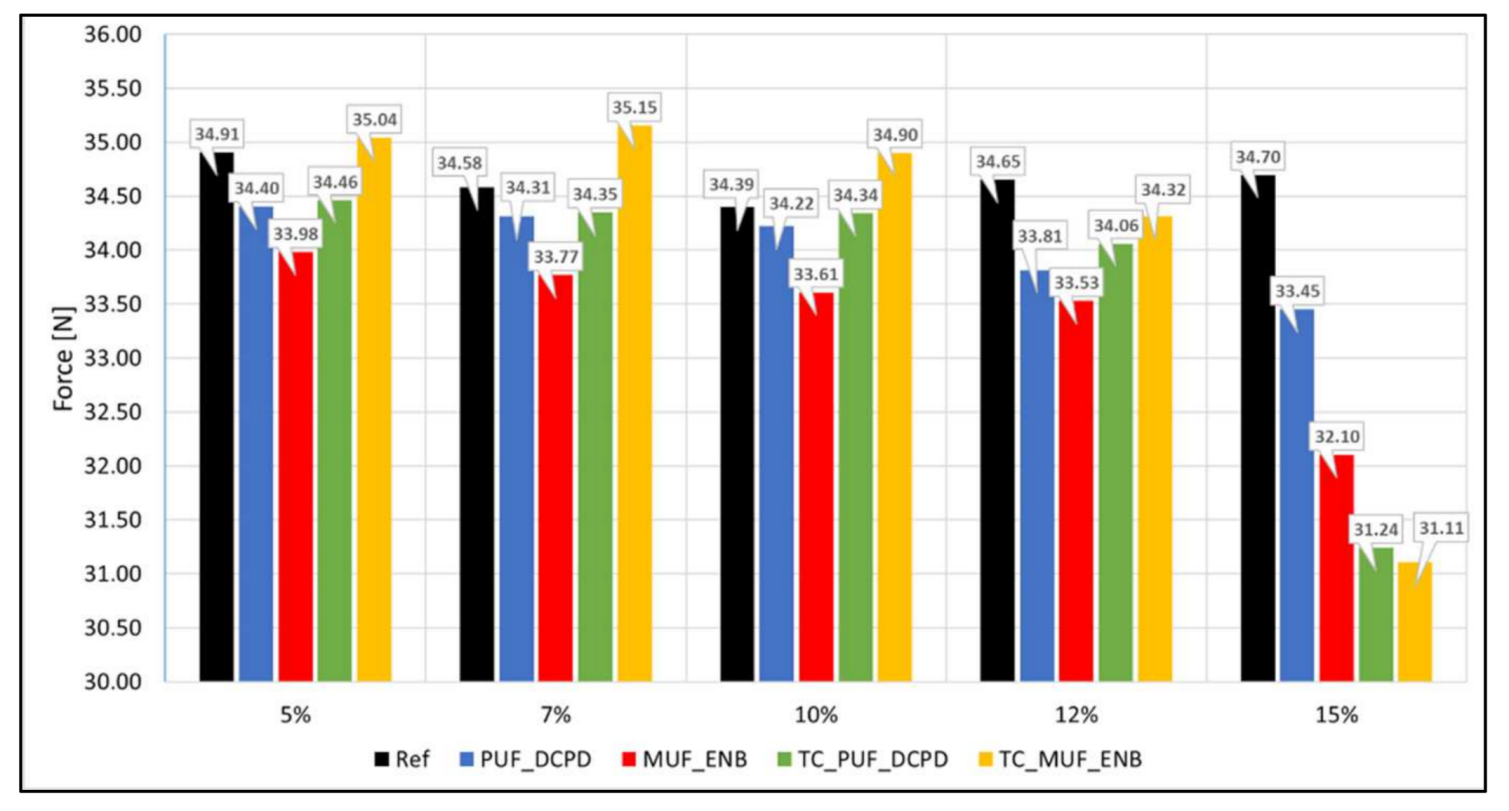
Impact strength with respect to microcapsule addition.

**Figure 11:**
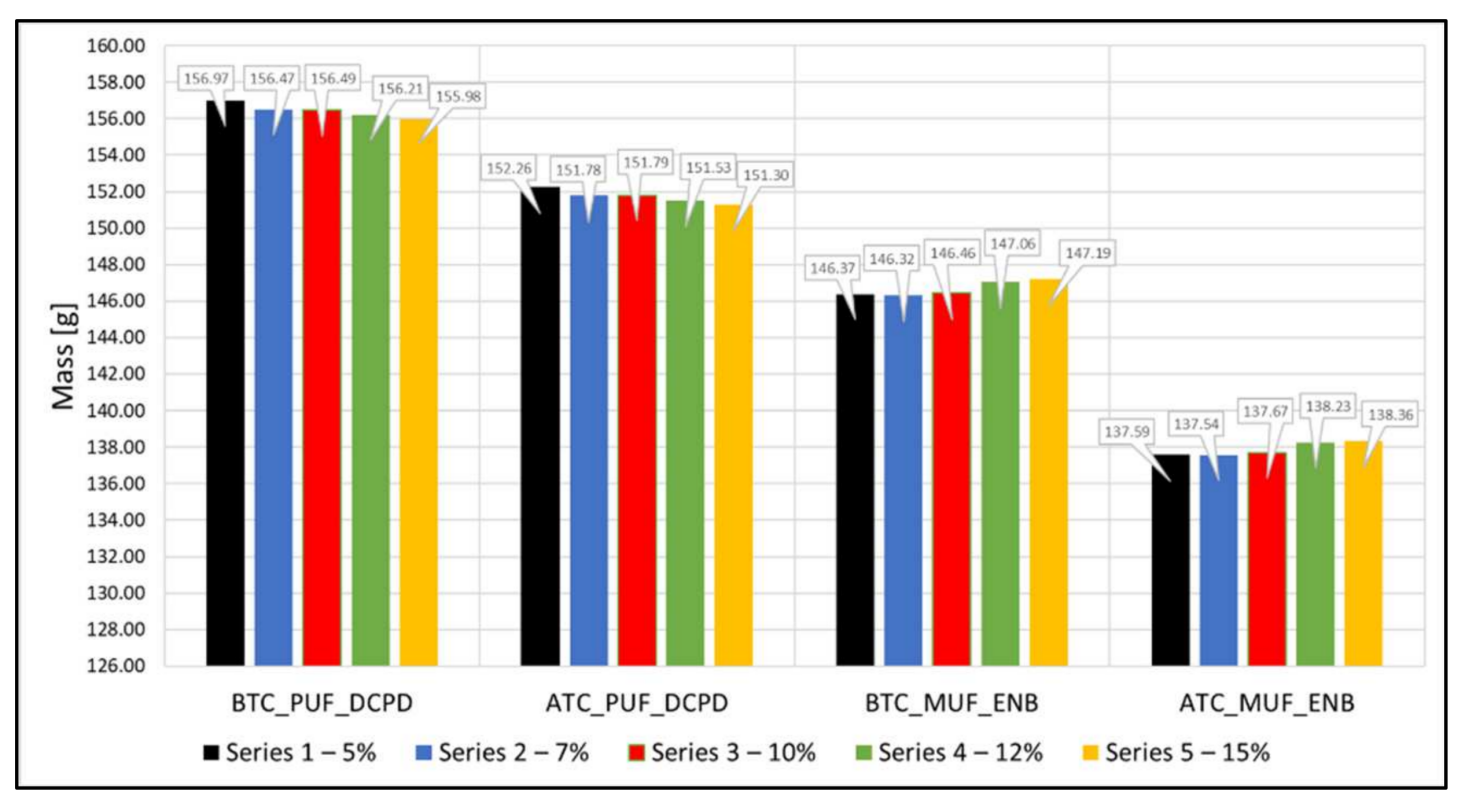
Mass loss after thermal cycling.

Figure 10 illustrates the influence of microcapsule volume addition on the composite impact strength. Increasing the microcapsule volume from 5% to 15% has a small effect on the impact strength of PUF-DCPD specimens, while for MUF-ENB specimens, the impact strength is even lower than for PUF-DCPD specimens, but following the same decreasing path. The difference between reference and ENB specimens is only 0.37% for 5% microcapsule addition and 1.5% for 7% microcapsule addition. These extremely low increasing values can be considered negligible as the small volume does not influence the impact strength and that they are thermally stable (up to 250 °C) and do not indicate any mass loss, as seen also in Figure 11. DPCD specimens after thermal cycling also tend to have the same impact strength as the specimens not subjected to thermal cycling, indicating the thermal stability of such systems. The specimens with 15% microcapsule addition present the lowest impact strength for both healing systems.

The mass of each specimen was measured before (BTC) and after thermal cycles (ATC), and is presented in Figure 11. It was observed that the average mass loss after thermal cycling was about 3% for PUF-DCPD specimens and about 6% for MUF-ENB specimens, which can be due to the degradation of healing agent components and some unreacted epoxy groups from the lay-up process of the healing system.

### 3.4. Impact Area Analysis

**Figure 12:**
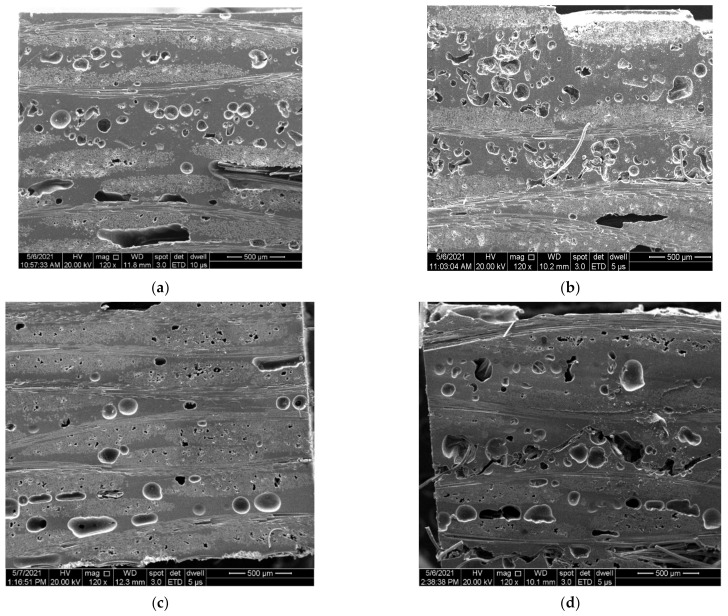
Representative SEM micrographs at 500 µm from impacted samples for (**a**) DCPD sample with 5% microcapsules; (**b**) DCPD sample with 15% microcapsules; (**c**) ENB sample with 5% microcapsules; (**d**) ENB sample with 15% microcapsules.

**Figure 13:**
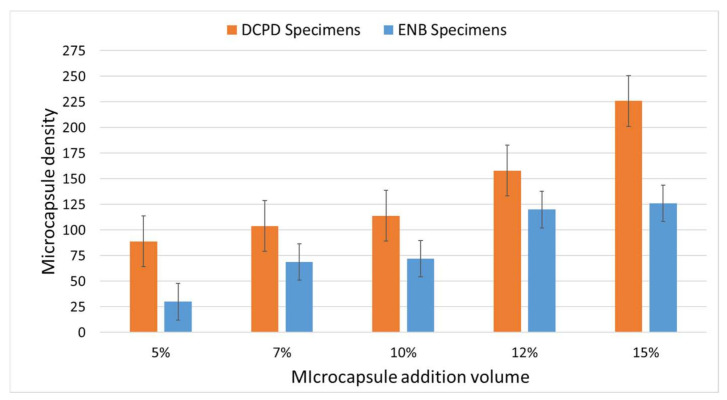
Total microcapsule density with respect to their volume addition.

SEM samples were taken after the second impact test. Samples were cut from near the impact area, as the samples that were initially cut from the impact area delaminated during sample preparation and could not be investigated. Representative SEM images of DCPD and ENB systems with 5% and 15% microcapsule volume are presented in Figure 12. The presence of some rupture-like areas is due to sample preparations (sample cut and polishing) prior to analysis. The samples analysed presented a homogenous dispersion of microcapsules for 5–12% volume addition. However, the sample containing 15% microcapsule volume showed some local agglomerations, which could be the reason for the impact force drop presented in Figure 10. These local agglomerations may be caused during the pouring of the resin and microcapsules mixture at the time of specimen manufacturing. The density of the microcapsules volume for both systems was measured over different areas of the samples and is presented in Figure 13.

**Figure 14:**
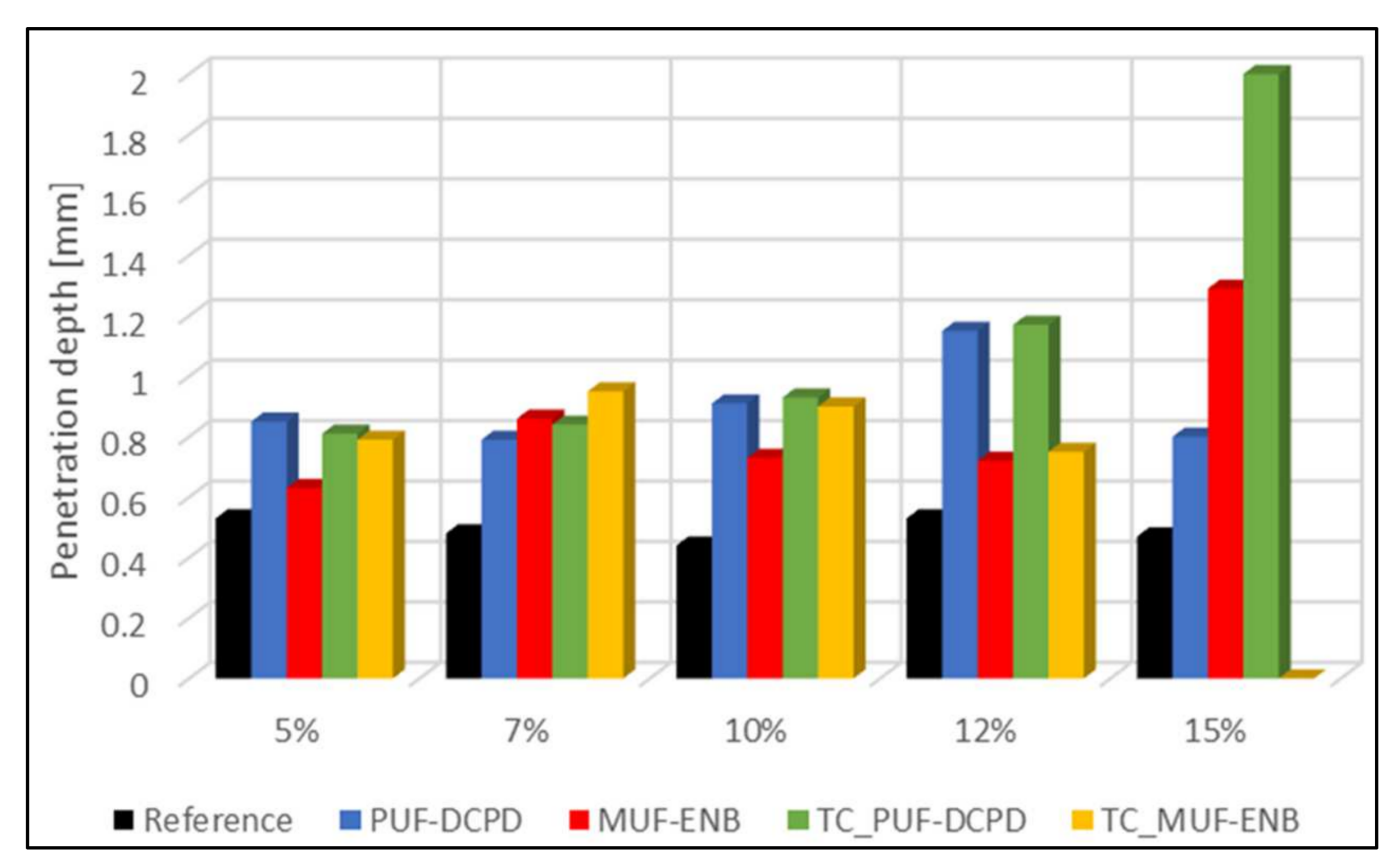
Penetration depth vs. microcapsule addition.

Knowing that the addition of self-healing systems to the composite structure acts as an induced defect, the objective of this analysis was to highlight the behaviour of each self-healing system and the influence on impact resistance (penetration depth) depending on the percentage of microcapsules added (Figure 14). The analysis was performed only after the second impact test. As can be observed from Figure 14, the specimen containing 15% MUF-ENB and subjected to thermal cycling was penetrated to its full thickness. A correlation between Figure 9 and Figure 10 can be made, as the 15% addition of microcapsules subjected to thermal cycling drastically decreases the impact strength, up to almost complete penetration of DCPD specimen (total specimen thickness is 2 mm) and to a total penetration of ENB specimens.

## 4. Discussion

The process of integrating the two self-healing systems (PUF-DCPD and MUF-ENB) in the Resoltech 1050/1058 epoxy system was investigated, using two dispersion methods, magnetic stirring and sonication. Thus, following microscopic analyses, it was found that dispersion by magnetic stirring offers a better homogeneity of the self-healing systems, while the ultrasonic waves destroy some of the microcapsules during the homogenization process. As the investigations in this paper were performed with certain sonication parameters and a specific instrument, the results conclude that magnetic stirring, at least for the presented findings, is the most appropriate method of dispersing microcapsules. However, using other equipment with other process parameters, in terms of time, pulsation and amplitude, could lead to a more homogenous dispersion without damaging the microcapsule. Another factor to consider is the equipment probe. Using a more sensitive probe, or one typical for this application, could lead to better results of sonication dispersions. It has also been observed that microcapsules tend to rise to the surface of specimens during the polymerization processes.

Three-point bending tests were performed to further evaluate the dispersion methods. Following these tests and the microstructural analysis, it was concluded that the dispersion by magnetic stirring was the optimum dispersion method.

As discussed by [12], the impact energy is an important factor influencing the effect of healing, meaning that with an increase in impact energy, the healing efficiency decreases due to the change in the dominant damage mode from matrix cracking to delamination and fibre rupture. Yin [12] performed compression after impact tests on woven fibreglass reinforced epoxy composites impacted up to 3.5 J, showing improved healing performance at lower impact energies. A 50% recovery in strength was found by [27], after performing a Charpy test on glass fibre specimens containing 7.5% microcapsule volume. For PUF-DCPD specimens, the use of 10% microcapsules, indicated an 84% recovery of impact strength, as compared to values indicated by [13,15]. It was also reported in [1] that a maximum damage recovery was observed for 10% microcapsule addition for the impact energy of 45 J. Moreover, the use of 15% microcapsules shows a slight deviation of the impact behaviour compared to the reference specimens. However, the maximum impact force is not much influenced by the volume of microcapsules introduced, as can also be seen in Figure 10. For the MUF-ENB specimens, the impact behaviour tends to decrease with microcapsule addition, and a higher drop in impact strength was recorded for 15% microcapsule addition. The thermal cycling did not influence the impact strength of PUF-DCPD specimens as compared to specimens not exposed to temperature variation, as it would be expected and it was again observed that the 15% addition of microcapsules decreases the impact strength by 7% compared to the specimen not exposed to temperature variation. However, different behaviour was observed for specimens containing MUF-ENB microcapsules, as the impact strength increased with microcapsule addition, after thermal cycling. The only difference was observed for the 15% addition of MUF-ENB microcapsules volume, where the impact strength decreased severely.

It can also be said that positive results were obtained for the specimens containing small volumes of microcapsules, 5%, 7% and 10%, with a percentage of 80–88% of the impact force after retesting, compared to the initial value. For PUF-DCPD specimens, the use of 12% microcapsules shows a slight deviation from the impact behaviour of the reference specimen, this being similar to the specimen with 12% MUF-ENB healing system.

For specimens with a 15% volume of microcapsules, the values of the impact forces were on average reduced by 60%, which indicates that an agglomeration of microcapsules on the small surface is not favourable. In the case of specimens subjected to thermal cycles, the most unfavourable results were obtained for specimens with a large volume of microcapsules.

The three-dimensional analysis showed that PUF-DCPD specimens had the highest penetration depth for 12% microcapsule addition, while MUF-ENB specimens for 15% volume. Testing of specimens subjected to thermal cycling showed that the PUF-DCPD specimen with 15% microcapsules had the highest penetration depth, as expected, while the MUF-ENB specimen has failed, being fully penetrated.

## 5. Conclusions

Within this work, an evaluation of the two different dispersion methods was performed to identify optimal processes for both PUF-DCPD and MUF-ENB systems, with respect to their integration in the host polymeric (epoxy) matrix. Three-point bending tests and microstructural analysis were performed to evaluate the two integration methods.

FT-IR was conducted to validate the presence of constitutive elements for the two healing systems. Additionally, the thermogravimetric analysis showed thermal degradation of 80% for the PUF-DCPD system and 82% for MUF-ENB.

Different volumes of the two healing systems (5%, 7%, 10%, 12% and 15%) were embedded using GFRP to evaluate the impact behaviour of microcapsule addition. Test specimens containing all microcapsules volumes addition were subjected to thermal cycling between −20 °C and +100 °C. It was concluded that microcapsule volume has little to no effect on the impact force; however, subjecting the specimens to temperature variation indicated a decrease in impact force, especially for the 15% microcapsule addition, for both healing systems.

## Figures and Tables

**Table 1:** Materials used in the synthetization of self-healing PUF-DCPD microcapsules.

Material	Molecular Formula	Physical Properties	Role
Urea	CH_4_N_2_O	Crystalline, white powder. Melting point at 133–135 °C	Formation of the capsule shell in the aqueous state
Resorcinol (1,3-benzenediol)	C_6_H_4_(OH)_2_	White crystals. Melting point at 113 °C	Blending resin with formaldehyde
Formaldehyde	CH_2_O	Colourless aqueous solution	Formation of capsule shell
Dicyclopentadiene (DCPD)	C_10_H_12_	Solid state (gel-like state at room temperature). Melting point at 32.5 °C	Monomer-capsule core
Maleic anhydride	C_2_H_2_(CO)_2_O	Solid state. White powder	Emulsifier
Ammonium chloride	NH_4_Cl	Solid state. White powder	Formaldehyde hardener
Sodium hydroxide	NaOH	Solid state. White powder	Rising solution pH
Hydrochloric acid	HCl	Aqueous solution with a strong odour	Lowering solution pH

**Table 2:** Materials used in the synthetization of self-healing MUF-ENB microcapsules.

Material	Molecular Formula	Physical Properties	Role
Melamine	C_3_H_6_N_6_	White powder. Melting point at 345 °C	Formation of the capsule shell
Sodium lauryl sulfate (SLS)	CH_3_(CH_2_)_10_CH_2_ (OCH_2_CH_2_)_n_OSO_3_Na	Powder soluble in water	Emulsifier—oil solidification
Polyvinyl alcohol (PVA)	(C_2_H_4_O)_x_	Melting point at 200 °C	Separation film
Urea	CH_4_N_2_O	Crystalline white powder. Melting point at 133–135 °C	Formation of the capsule shell in the aqueous state
Formaldehyde	CH_2_O	Colourless aqueous solution	Formation of the capsule shell

**Table 3:** Variation of sonication parameters.

Nr. Crt.	Parameters	Results	Observation
1	Amplitude: 20%	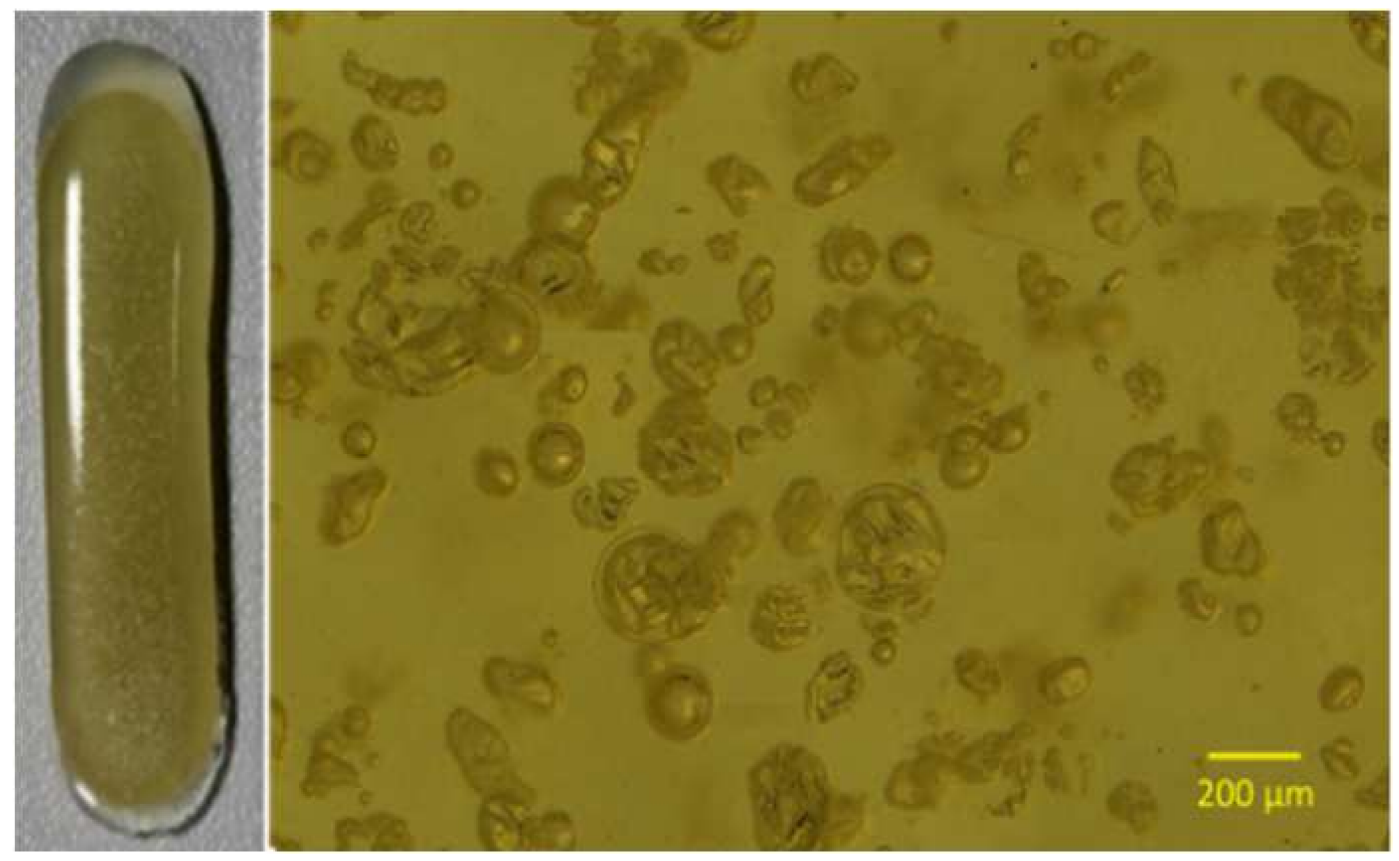	A small homogenization of microcapsules was observed for sonication with 20% amplitude for 60 s. This can be due to the short sonication time and low amplitude. As the sonication time increases, a better homogenization was observed.
	Pulsation: 10 s		
	Off time: 2 s		
	Total time: 60 s		
2	Amplitude: 20%	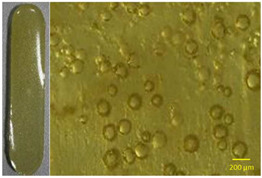	
	Pulsation: 10 s		
	Off time: 2 s		
	Total time: 600 s		
3	Amplitude: 40%	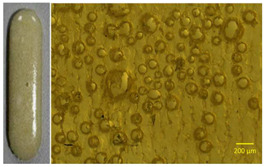	Increasing the amplitude to 40% resulted in better dispersion of the microcapsules, but the short sonication period used to microcapsule agglomeration. Increasing both the amplitude and the total sonication time, resulted in a good homogenization of the microcapsules.
	Pulsation: 10 s		
	Off time: 2 s		
	Total time: 60 s		
4	Amplitude: 40%	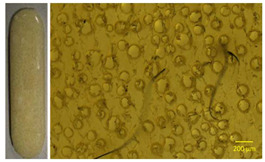	
	Pulsation: 10 s		
	Off time: 2 s		
	Total time: 600 s		
5	Amplitude: 60%	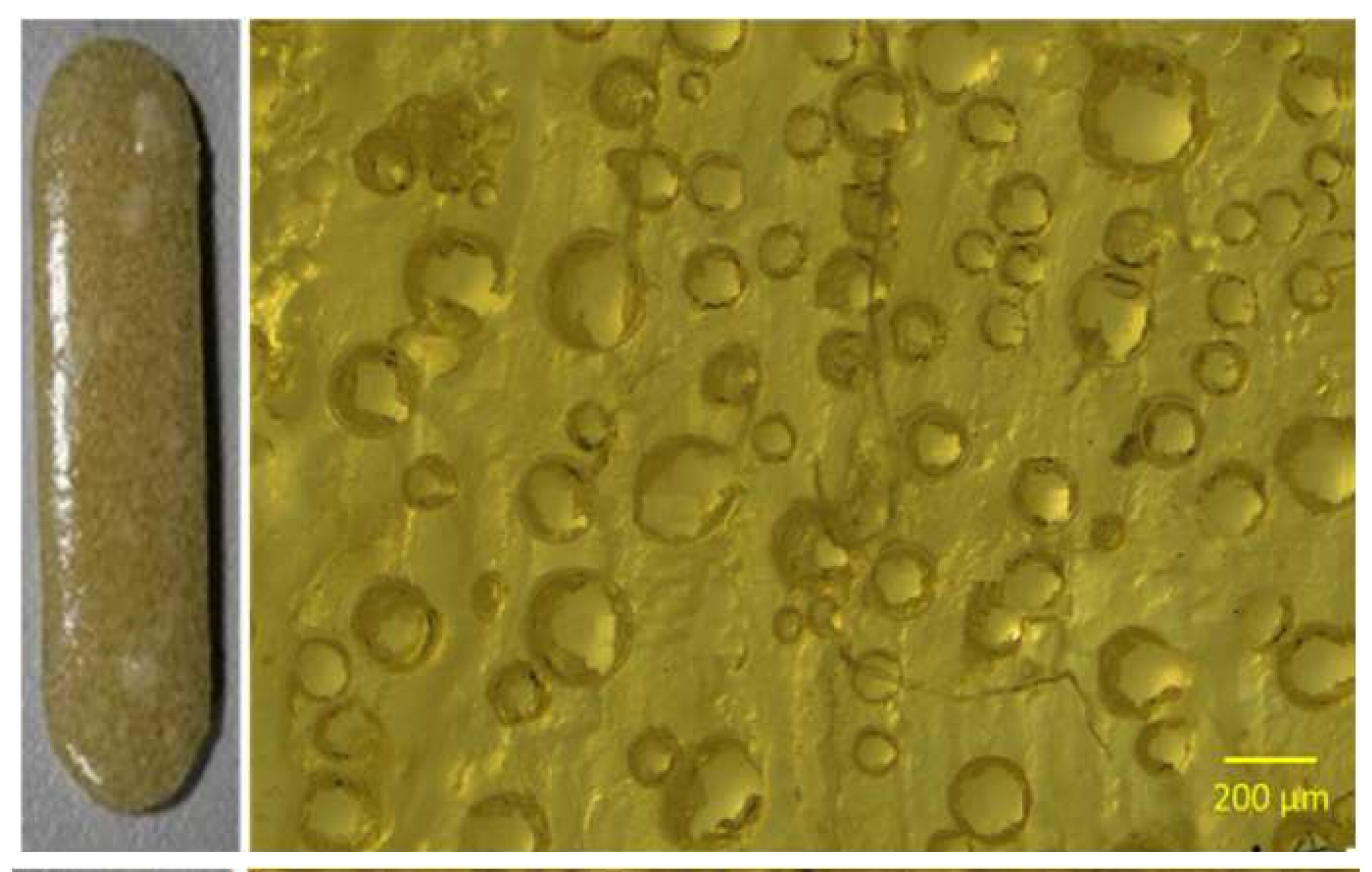	Further increasing the amplitude and sonication time led to microcapsule rupture and heterogeneous dispersion.
	Pulsation: 10 s		
	Off time: 2 s		
	Total time: 60 s		
6	Amplitude: 60%	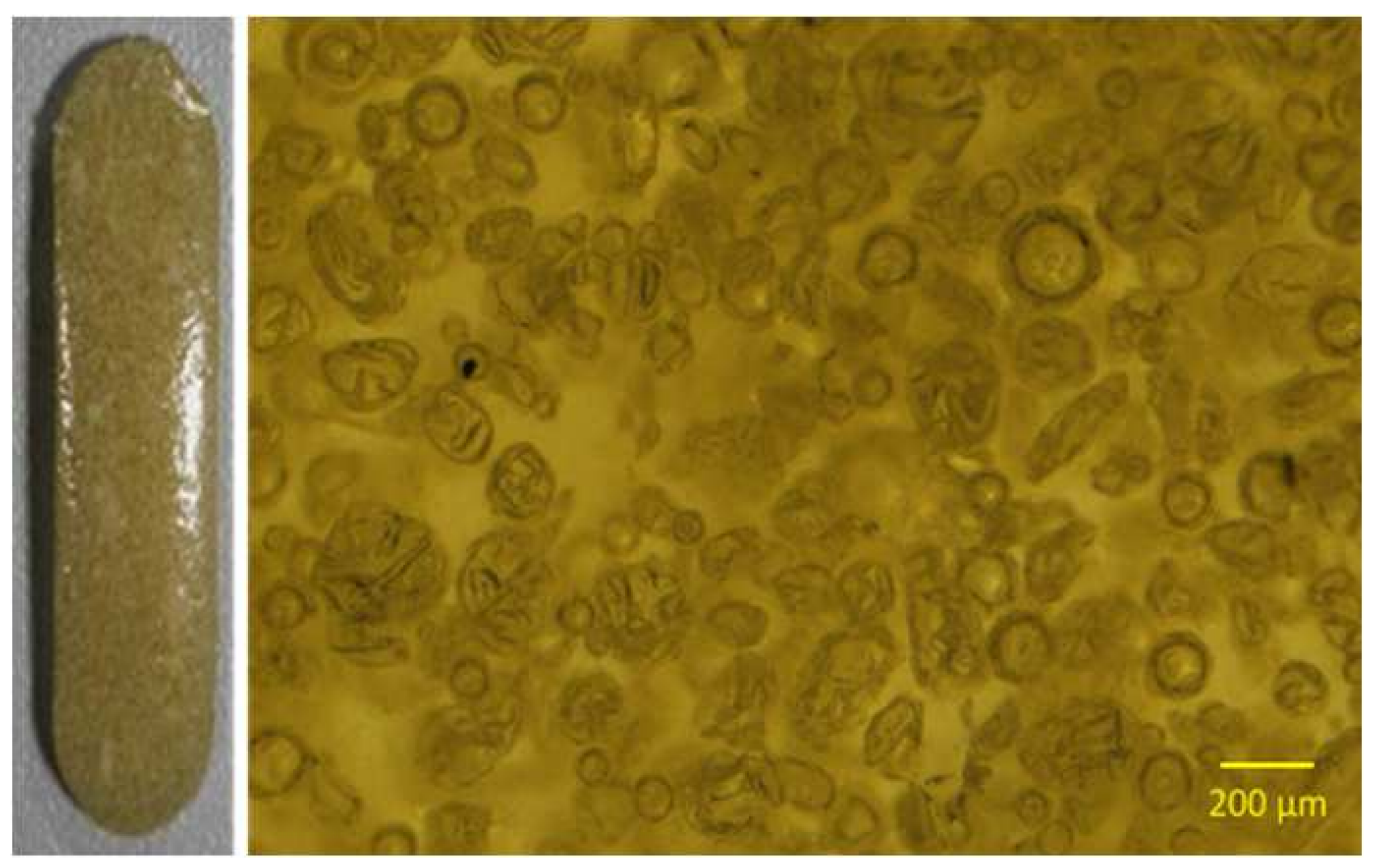	
	Pulsation: 10 s		
	Off time: 2 s		
	Total time: 600 s		

**Table 4:** Impact specimen configurations.

Specimen	**Specimen ID**				
	**Ref_1**	**Ref_2**	**Ref_3**	**Ref_4**	**Ref_5**
	DCPD_1	DCPD_2	DCPD_3	DCPD_4	DCPD_5
	ENB_1	ENB_2	ENB_3	ENB_4	ENB_5
	TC_DCPD_1	TC_DCPD_2	TC_DCPD_3	TC_DCPD_4	TC_DCPD_5
	TC_ENB_1	TC_ENB_2	TC_ENB_3	TC_ENB_4	TC_ENB_5
Microcapsules	5%	7%	10%	12%	15%
Catalyst	2%	2.5%	4%	4.5%	6%

Where Ref is Reference specimen; DCPD is the specimen with PUF-DCPD self-healing system; ENB is the specimen with MUF-ENB self-healing system; TC is Thermal Cycling.

**Table 5:** Crosslink density for the two self-healing systems.

Sample	Tg (°C)	Crosslinking Density (v_e_)	Storage Modulus (MPa)	Loss Modulus (MPa)
Reference	88.1	3098	3789.05	57.48
S1	80.5	5092	2892.66	59.29
S2	70.2	3149	4130.25	59.61

**Table 6:** Three-point bending results comparison for the two dispersion methods.

		Flexural Strength	Load	Elongation at Break
		Average [MPa]	Average [MPa]	Average [MPa]
Neat epoxy specimens		63.01	41.50	16.83
PUF-DCPD system	Magnetic stirring	58.56	39.05	18.34
	Sonication	52.70	34.85	20.73
MUF-ENB system	Magnetic stirring	55.73	3.46	20.59
	Sonication	50.35	34.09	22.65

Where St.dev. represents the standard deviation.

**Table 7:** Three-point bending flexural modulus and absorbed energy results.

	Reference		DCPD Magnetic		DCPD Sonication		ENB Magnetic		ENB Sonication	
	E [MPa]	AE [J]	E [MPa]	AE [J]	E [MPa]	AE [J]	E [MPa]	AE [J]	E [MPa]	AE [J]
Specimen 1	3325.72	4062.69	2447.37	3430.54	3513.05	2280.84	2416.34	3038.26	3039.09	2792.08
Specimen 2	3704.74	3963.80	3569.16	2742.37	3575.05	2496.05	2978.9	3167.35	3549.13	2372.28
Specimen 3	3309.20	3126.31	3002.83	2153.04	3864.28	2805.59	2731.06	3034.97	3196.19	2292.53
Specimen 4	5679.64	4316.23	2364.42	3535.88	3350.56	2926.37	2694.80	3115.84	3037.63	2890.93
Specimen 5	3835.18	3795.85	3034.49	2412.54	2757.11	2656.36	3278.13	2862.83	3275.54	2610.37

## Data Availability

No new data were created or analyzed in this study. Data sharing is not applicable to this article.
